# Medical Classes

**Published:** 1852-03

**Authors:** 


MEDICAL CLASSES.

The New York Medical Gazette says, that the highest numbers counted on the benches in the various medical schools of that city are as follows: "In the College of Physicians and Surgeons, 197; in the University of the city of New York, 179; in the New York Medical College, 69."

The number of *bona fide* students in attendance on the course of lectures in the medical department of the University of Louisville, just closed, was 261.

